# Neutrophil‐lymphocyte ratio in relation to risk of hepatocellular carcinoma in patients with non‐alcoholic fatty liver disease

**DOI:** 10.1002/cam4.5185

**Published:** 2022-09-02

**Authors:** Claire E. Thomas, Yi‐Chuan Yu, Hung N. Luu, Renwei Wang, Pedram Paragomi, Jaideep Behari, Jian‐Min Yuan

**Affiliations:** ^1^ Cancer Epidemiology and Prevention Program University of Pittsburgh Medical Center (UPMC) Hillman Cancer Center, University of Pittsburgh Pittsburgh Pennsylvania USA; ^2^ Department of Epidemiology, Graduate School of Public Health University of Pittsburgh Pittsburgh Pennsylvania USA; ^3^ Department of Medicine, Division of Gastroenterology, Hepatology, and Nutrition University of Pittsburgh Pittsburgh Pennsylvania USA

**Keywords:** electronic health records, hepatocellular carcinoma, non‐alcoholic fatty liver disease

## Abstract

**Background:**

Blood neutrophil to lymphocyte ratio (NLR) or lymphocyte count may be important markers for immune function. Previous work has shown higher NLR was associated with higher risk of hepatitis B‐related hepatocellular carcinoma (HCC). However, studies in non‐alcoholic fatty liver disease (NAFLD) patients are lacking.

**Methods:**

Utilizing the University of Pittsburgh Medical Center (UPMC) electronic health records, we created a retrospective cohort of 27,834 patients diagnosed with NAFLD from 2004 to 2018 with complete NLR data. After an average 5.5 years of follow‐up, 203 patients developed HCC. Cox proportional hazard regression was used to calculate hazard ratios (HRs) and 95% confidence intervals (CIs) of HCC incidence associated with different levels of NLR and lymphocyte count.

**Results:**

Compared with the lowest tertile of NLR (<1.97), the highest tertile of NLR (≥3.09) was statistically significantly associated with a 43% higher risk of HCC incidence (HR = 1.43, 95% CI: 1.01–2.03, *p*_trend_ = 0.031) after adjustment for age, sex, race, body mass index, smoking status, history of type 2 diabetes, hyperlipidemia, hypertension, and fibrosis‐4 score category. Conversely the highest tertile of lymphocyte count (≥2.15 K/ul) was significantly associated with a 36% lower risk of HCC (HR = 0.64, 95% CI: 0.43–0.94, *p*_trend_ = 0.028) compared to the lowest tertile (<1.55 K/ul). There was no association between neutrophil count and HCC risk.

**Conclusions:**

Higher NLR and lower lymphocyte count are associated with significantly higher risk of HCC among NAFLD patients. These findings warrant further investigation of immune response and surveillance in association with HCC development in NAFLD patients.

## Graphical abstract

Utilizing the University of Pittsburgh Medical Center (UPMC) electronic health records, we found that higher neutrophil‐to‐lymphocyte ratio and lower absolute lymphocyte count were associated with higher risk of hepatocellular carcinoma in a cohort of non‐alcoholic fatty liver disease patients. Future studies of the impact of immune response and surveillance through neutrophil‐lymphocyte ratio or lymphocyte count on hepatocellular carcinoma incidence are warranted.

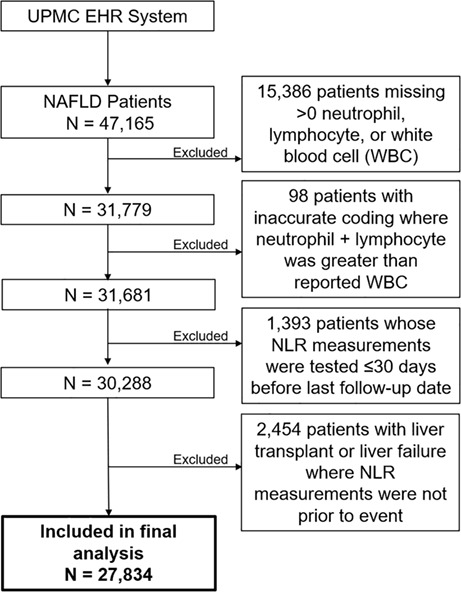

## 1 INTRODUCTION

Primary liver cancer is an emerging public health problem. Global deaths due to primary liver cancer are predicted to reach 1 million by 2030.^1^, ^2^ Hepatocellular carcinoma (HCC) is the major subtype of primary liver cancer.^3^ Approximately 75%–90% of total primary liver cancer cases are HCC.^4^ There are large geographical disparities of HCC cases: the highest incidence rate are found in sub‐Saharan Africa and Eastern Asia (>20.0 per 100,000 persons) while the lowest is found in North America and Northern Europe (<5.0 per 100,000 persons).^5^ In the U.S., the major risk factors for HCC includes chronic infection with hepatitis B virus (HBV) and/or hepatitis C virus (HCV), which account for approximately 50% of all HCC cases.^6^ Other established risk factors for HCC development include heavy alcohol use, cigarette smoking, and in certain high‐risk regions, exposure to dietary aflatoxin.^7^, ^8^, ^9^, ^10^

Non‐alcoholic fatty liver disease (NAFLD) and its related conditions such as obesity and diabetes is recognized as an emerging risk factor for HCC,^11^ and is also one of the most common chronic liver conditions in most developed nations.^12^ The global prevalence of NAFLD is 30%–40% in men and 15%–20% in women.^13^ NAFLD includes a wide range of liver disorders from simple steatosis and nonalcoholic steatohepatitis to fibrosis and cirrhosis which may eventually progress to HCC.^14^, ^15^, ^16^, ^17^ Only a small proportion (1.2%) of the early‐stage NAFLD may progress to cirrhosis,^18^ and the incidence rate of HCC was around 1% per year among NAFLD patients with cirrhosis.^19^ Biomarkers that may be related to the progression of NAFLD to HCC are understudied. Neutrophil‐lymphocyte ratio (NLR) has previously been found to be associated with the prognosis for patients with HCC, colorectal cancer, or pancreatic cancer^20^, ^21^, ^22^ and has also been proposed as a prognostic marker for late‐stage liver conditions such as cirrhosis^23^ and fibrosis.^24^ However, studies of NLR association with HCC risk are sparse, especially in an at‐risk NAFLD population. NLR may be a marker of inflammation related to tumor initiation through sustained neutrophil stimulation.^25^ Alternatively reduced lymphocyte count may be reflective of reduced immune surveillance capacity, which could lead to the escape of malignant cells and tumor growth.^26^ The focus of the present analysis for NLR and HCC risk in a NAFLD patient population may avoid potential confounding by chronic HBV/HCV infection or auto‐immune disease, which are likely to cause changes in leukocyte counts in circulation with known plausible pathological mechanisms. Our analysis attempts to shed light on the mechanism between NLR levels and HCC risk in a more homogeneous patient population (i.e. NAFLD).

In the present study, we examined the association for absolute neutrophil count, absolute lymphocyte count, and NLR with risk of HCC among a large cohort of NAFLD patients otherwise absent of established major risk factors for HCC such as viral hepatitis, alcoholic liver disease, and autoimmune liver disease derived from the electronic health records (EHR) in a large healthcare network system in western Pennsylvania, USA.

## 2 METHODS

### 2.1 Study population

We conducted a retrospective cohort study of participants of the University of Pittsburgh Medical Center (UPMC) Health Insurance Plan with NAFLD. UPMC includes 40 hospitals and 700 doctors' offices and outpatient sites that serve more than 3 million patients annually throughout western Pennsylvania, USA. Data was requested through the Health Record Research Requestion (R3) provided by the Biomedical Informatics Services at the University of Pittsburgh. In the present analysis, we requested deidentified EHRs of all UPMC Health Plan participants, aged 40–89 years old, who ever used medical service at UPMC Health Network System from 1/1/2004 to 12/31/2018. The study was approved by the University of Pittsburgh Internal Review Board.

### 2.2 Inclusion and exclusion criteria for the NAFLD cohort

Eligibility criteria for the NAFLD cohort study were patients with any of the following diagnoses according to the International Classification of Diseases version 9 (ICD‐9) or 10 (ICD‐10) codes on the EHRs: non‐alcoholic fatty liver (NAFL), non‐alcoholic steatohepatitis (NASH), cirrhosis of liver without mention of alcohol, hepatic failure, or hepatic encephalopathy (Table S1). Cirrhosis was defined based on ICD codes for either compensated or decompensated cirrhosis (Table S1). Exclusion criteria for the NAFLD cohort were patients with any of the following diagnoses based on their ICD‐9 or ICD‐10 codes on the EHRs: alcoholic liver disease, alcohol use disorder, somatic consequences of alcohol, autoimmune liver disease, alpha‐1‐antitrypsin deficiency, secondary or unspecified biliary cirrhosis, drug use disorder except nicotine/caffeine, hemochromatosis, Budd‐Chiari syndrome, viral hepatitis, unspecified chronic hepatitis, or Wilson's disease (**Table** **S2**). These inclusion and exclusion criteria were based on the recent consensus statement by an expert panel for the definition of NAFLD using administrative coding in electronic health care records.^27^ The UPMC NAFLD Cohort Study consisted of 47,165 patients.

**FIGURE 1:**
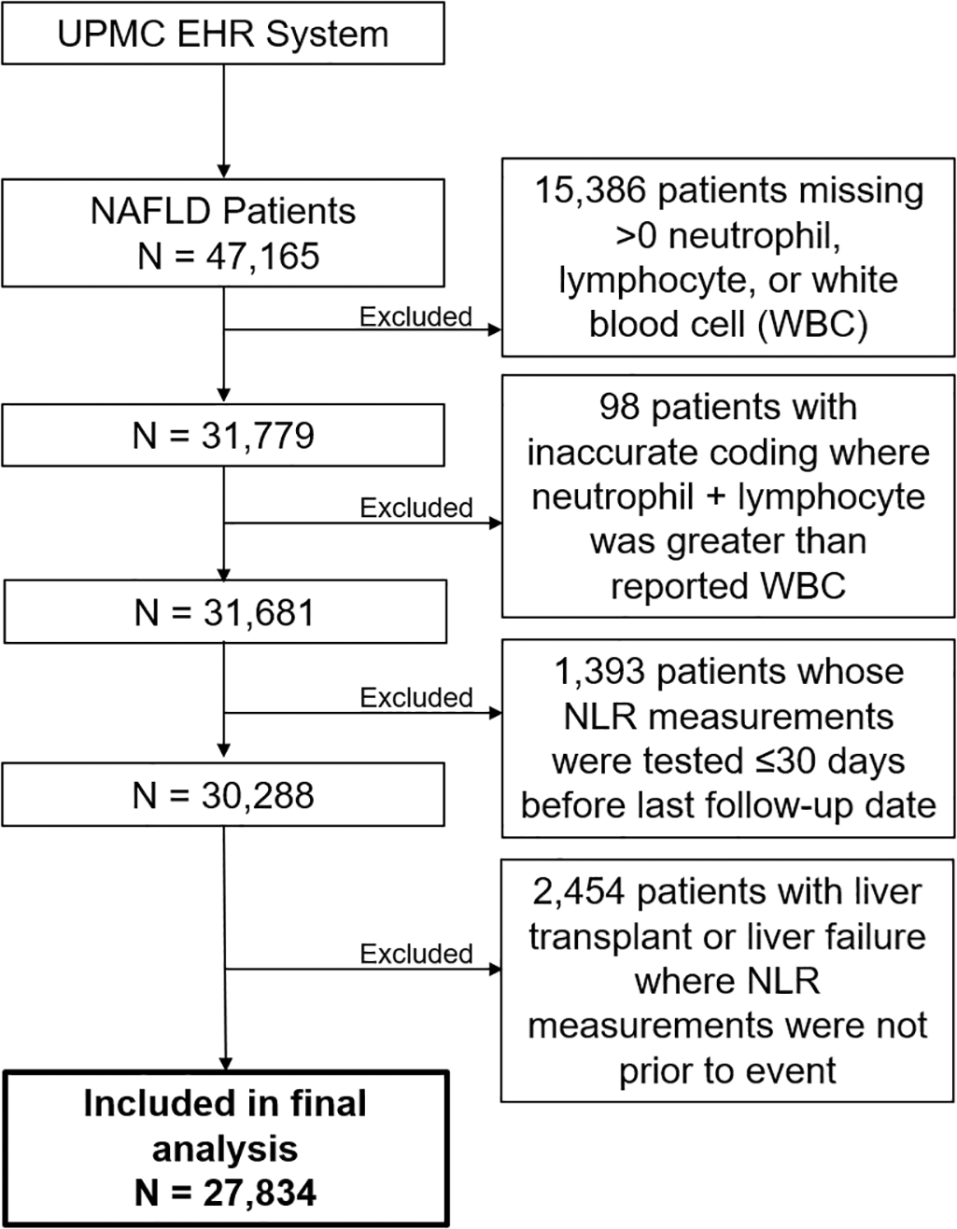
Construction of the neutrophil‐lymphocyte ratio and the risk of HCC final analytic dataset from the UPMC NAFLD Cohort Study, 2004–2018. EHR, electronic health record; NAFLD, non‐alcoholic fatty liver disease; NLR, neutrophil‐lymphocyte ratio; UPMC, University of Pittsburgh Medical Center; WBC, white blood cell.

The primary exposures of interest in the present study were absolute neutrophil count (K/ul) and absolute lymphocyte count (K/ul). The neutrophil to lymphocyte ratio (NLR) was calculated by using these two absolute counts. Of the 47,165 NAFLD patients, 15,386 were excluded for missing data on neutrophil, lymphocyte, or white blood cell count measurements; 98 were excluded due to data errors resulting in the sum of neutrophil and lymphocyte counts greater than the total white blood cell counts. 1393 patients were excluded because the white blood cell counts were measured less than or equal to 30 days prior to the last date of follow‐up and 2454 patients were excluded because of the lack of white blood cell counts before they underwent liver transplantation or liver failure (Figure 1). Covariates extracted from the EHRs include age, sex, history of type II diabetes, race, body mass index (BMI), smoking status, hyperlipidemia status, hypertension status, and Fibrosis‐4 (FIB‐4) score category. FIB‐4 score, a a biomarker of liver fibrosis, was calculated by an established formula as follows^28^: $\frac{\mathrm{Age}\;\left(\text{years}\right)\times \mathrm{AST}\;\left(U/L\right)}{\text{Platelet Count}\;\left(10^{9}/L\right)\times \surd \mathrm{ALT}\left(U/L\right)}.$ The biomarker components of FIB‐4 score — platelet count, aspartate aminotransferase (AST), and alanine aminotransferase (ALT) — were obtained from the EHR with their measurement dates that were closest to the measurement of white blood cell counts. Any of the measurements that was done within 30 or less days prior to HCC diagnosis or death was treated as missing to minimize potential bias by disease status. FIB‐4 score was categorized as <1.30 (<65 years old)/<2.00 (65+ years old) (referred as no/low liver fibrosis), 1.30–2.67 (<65 years)/2.0–2.67 (65+ years) (intermediate fibrosis), and >2.67 (advanced fibrosis). A separate category was created for those with missing FIB‐4 score.

Age was calculated for time of white blood cell count date and history of type II diabetes, hyperlipidemia, and hypertension included all diagnoses up to WBC measurement.

The outcome of interest in the present study was incident HCC with the initial diagnosis date from January 1, 2004, through December 31, 2018. All HCC cases were identified based on ICD‐9‐CM code 155.0 or ICD‐10‐CM codes C22.0 and C22.8. After exclusions, 27,834 patients including 203 HCC cases were included in the present study.

### 2.3 Statistical analysis

Person‐years at risk for each patient were calculated from the date of neutrophil and lymphocyte measurement to the date of HCC diagnosis, death, or last encounter with any UPMC Healthcare Network facility, whichever occurred first. Tertiles of the absolute neutrophil count, absolute lymphocyte count, and NLR were derived according to their distributions among the 27,834 patients included. Chi‐square test was used to examine the frequencies of categorical variables by the tertile levels of NLR or HCC status whereas Wilcoxon two‐sample test or Kruskal‐Wallis test was used for continuous variables. The distributions of absolute neutrophil and lymphocyte counts and NLR were rightward skewed, therefore medians and 25th, 75th percentiles are presented for differences between HCC cases and non‐cases.

Cox proportional hazards regression models were used to estimate hazard ratios (HRs) and 95% confidence intervals (CIs) for HCC associated with tertile and doubling (log_2_) of neutrophil, lymphocyte, or NLR measurements with adjustment for potential confounding factors. Initial models were adjusted for only age (years) and sex (male, female). Additional covariates included in the Cox model were history of type II diabetes (yes, no), race (i.e., white, non‐white, missing), BMI category (i.e., underweight, normal weight, overweight, obese, or missing), smoking status (i.e., never smoker, former smoker, current smoker, unknown or passive smoke exposure, missing), hyperlipidemia status (yes, no), hypertension status (yes, no), and FIB‐4 score category. The linear trend for HCC risk with levels of neutrophils, lymphocytes, or NLR was tested based on the ordinal values of their tertiles. *p* value for interaction between NLR and a given variable for stratifying subgroups (e.g., sex or cirrhosis status) on HCC risk was derived from the product term of NLR multiplied by the stratifying variable included in the Cox proportional hazards regression model that also included both main terms – NLR and the stratifying variable, as well as other covariates. A similar approach was applied to lymphocyte or neutrophil count. In supplementary analyses time‐dependent Cox proportional hazards regression was used to investigate if the repeated measurements of white blood cells would provide similar results as the baseline measurement. An average of all repeated measurements available was calculated for each 12‐month period per patient following the baseline measurement for a maximum of 5 years of repeated measurements.

Statistical analyses were performed using SAS version 9.4 (SAS Institute Inc.). All *p* values presented are two‐sided, and *p* values less than 0.05 were considered statistically significant.

## 3 RESULTS

After an average (standard deviation) of 5.5 (3.4) years of follow‐up of 27,834 patients with available measurement of both blood neutrophils and lymphocytes, 203 developed HCC. Those in the highest tertile of NLR were more likely to be older, male, white, less obese, and have higher FIB‐4 score category, histories of cirrhosis, type II diabetes and hypertension compared to the lowest tertile (Table 1). Those in the highest tertile were less likely to be never‐smokers and had a lower prevalence of hyperlipidemia compared to the lowest tertile.

**TABLE 1:** Baseline characteristics by neutrophil‐lymphocyte ratio, by tertile, The UPMC NAFLD Cohort Study, 2004–2018

Characteristics	1st tertile (<1.97)	2nd tertile (1.97to <3.09)	3rd tertile (≥3.09)	*p*
Number of Subjects	9260	9274	9300	
Age, years, median (25th, 75th pctl)	56 (48, 63)	56 (48, 64)	59 (50, 67)	<0.001
Female sex, *n* (%)	5704 (62)	5268 (57)	4835 (52)	<0.001
Race, *n* (%)				
White	8282 (89)	8700 (94)	8643 (93)	<0.001
Non‐white	951 (10)	544 (6)	603 (6)	
Missing	27 (0.3)	30 (0.3)	54 (0.6)	
Body mass index, kg/m^2^, *n* (%)				
Underweight (<18.5)	15 (0.2)	28 (0.3)	43 (0.5)	<0.001
Normal weight (18.5 to <25)	650 (7)	584 (6)	819 (9)	
Overweight (25 to <30)	2326 (25)	2147 (23)	2148 (23)	
Obese (30+)	6005 (65)	6235 (67)	5797 (62)	
Missing	264 (3)	280 (3)	493 (5)	
Smoking status, *n* (%)				
Never smoker	4971 (54)	4852 (52)	4468 (48)	<0.001
Former smoker	2337 (25)	2416 (26)	2456 (26)	
Current smoker	1203 (13)	1249 (13)	1108 (12)	
Unknown or passive smoke exposure	414 (5)	405 (4)	439 (5)	
Missing	335 (4)	352 (4)	829 (9)	
Type 2 diabetes, *n* (%)	2224 (24)	2368 (26)	2764 (30)	<0.001
Hypertension, *n* (%)	4448 (48)	4633 (50)	5018 (54)	<0.001
Hyperlipidemia, *n* (%)	3600 (39)	3365 (36)	3018 (32)	<0.001
Cirrhosis, *n* (%)	884 (10)	1031 (11)	1547 (17)	<0.001
FIB‐4 score (age < 65/age ≥ 65)				
<1.30/<2.00	6126 (66)	6409 (69)	5910 (64)	<0.001
1.30–2.67/2.00–2.67	1818 (20)	1616 (17)	1707 (18)	
>2.67	573 (6)	595 (6)	1110 (12)	
Unknown	743 (8)	654 (7)	573 (6)	

*Note*: Chi‐square test was used for categorical variables; Kruskal‐Wallis test was used for continuous variables.

Abbreviations: NAFLD, non‐alcoholic fatty liver disease; ptcl, percentile; UPMC, University of Pittsburgh Medical Center.

HCC cases were older, higher percentage of former smokers, and lower percentage of women and obesity (Table 2). There were significantly higher proportion of HCC cases with a higher FIB‐4 score or cirrhosis diagnosis, where 66% of HCC cases were cirrhotic and 25% had FIB‐4 score >2.67 compared to non‐cases where only 12% were cirrhotic and 8% had FIB‐4 score >2.67. HCC cases had significantly lower lymphocyte count and higher NLR than non‐cases (both *p's* < 0.001). The levels of neutrophil count were comparable between HCC cases and non‐cases.

**TABLE 2:** Baseline characteristics by hepatocellular carcinoma status, The UPMC NAFLD Cohort Study, 2004–2018

Characteristics	HCC cases	Non‐cases	*p*
Number of Subjects	203	27,631	
Age, years, median (25th, 75th pctl)	66 (59, 74)	57 (49, 65)	<0.001
Female sex, *n* (%)	96 (47)	15,711 (57)	0.006
Race, *n* (%)			
White	191 (94)	25,434 (92)	0.508
Non‐white	11 (5)	2087 (8)	
Missing	1 (0.5)	110 (0.4)	
Body mass index, kg/m^2^, *n* (%)			
Underweight (<18.5)	3 (1)	83 (0.3)	<0.001
Normal weight (18.5 to <25)	30 (15)	2023 (7)	
Overweight (25 to <30)	56 (28)	6565 (24)	
Obese (30+)	101 (50)	17,936 (65)	
Missing	13 (6)	1024 (4)	
Smoking status, *n* (%)			
Never smoker	83 (41)	14,208 (51)	<0.001
Former smoker	74 (36)	7135 (26)	
Current smoker	20 (10)	3540 (13)	
Unknown or passive smoke exposure	8 (4)	1250 (5)	
Missing	18 (9)	1498 (5)	
Type 2 diabetes, *n* (%)	63 (31)	7293 (26)	0.135
Hypertension, *n* (%)	105 (52)	13,994 (51)	0.760
Hyperlipidemia, *n* (%)	60 (30)	9923 (36)	0.060
Cirrhosis, *n* (%)	134 (66)	3228 (12)	<0.001
FIB‐4 score (age < 65/age ≥ 65)			
<1.30/<2.00	90 (44)	18,355 (66)	<0.001
1.30–2.67/2.00–2.67	45 (22)	5096 (18)	
>2.67	51 (25)	2227 (8)	
Unknown	17 (8)	1953 (7)	
Absolute neutrophil count, K/ul, median (25th, 75th pctl)	4.7 (3.6, 6.2)	4.6 (3.5, 6.2)	0.900
Absolute lymphocyte count, K/ul, median (25th, 75th pctl)	1.6 (1.1, 2.0)	1.8 (1.4, 2.3)	<0.001
Neutrophil‐lymphocyte ratio, median (25th, 75th percentiles)	3.0 (2.0, 4.6)	2.4 (1.8, 3.7)	<0.001

*Note*: Chi‐square test was used for categorical variables; Wilcoxon two‐sample test was used for continuous variables.

Abbreviations: NAFLD, non‐alcoholic fatty liver disease; ptcl, percentile; UPMC, University of Pittsburgh Medical Center.

After adjustment for age and sex, the highest tertile of NLR was associated with a statistically significant 55% higher risk of HCC (HR = 1.55, 95% CI: 1.10–2.19) compared with the lowest tertile (*p*_trend_ = 0.008, Table 3). Further adjustment for race, BMI category, history of type II diabetes, smoking status, history of hyperlipidemia, history of hypertension, and FIB‐4 score category slightly reduced the risk estimate, but remained statistically significant (HR = 1.43, 95% CI: 1.01–2.03, *p*_trend_ = 0.031). The highest tertile of lymphocyte count was associated with a statistically significant 36% lower risk of HCC (HR = 0.64, 95% CI: 0.43–0.94, *p* = 0.028) compared to the lowest tertile with adjustment for all covariates (Table 3). Doubling concentration of absolute lymphocyte count was associated with a 24% lower risk of HCC whereas doubling of NLR was associated with a 14% higher risk of HCC (Table 3). There was no significant association between absolute neutrophil count and HCC risk in any models presented.

**TABLE 3:** Association between neutrophil‐lymphocyte ratio, absolute neutrophil count, and absolute lymphocyte count and the risk of hepatocellular carcinoma, The UPMC NAFLD Cohort Study, 2004–2018

Exposure	Total No. subjects	Total No. person‐years	HCC Cases	HR (95% CI)^a^	HR (95% CI)^b^
Neutrophil‐lymphocyte ratio					
Categorical					
1st tertile (<1.97)	9260	52,774	51	1.00	1.00
2nd tertile (1.97 to <3.09)	9274	52,543	56	1.02 (0.70, 1.50)	1.03 (0.70, 1.51)
3rd tertile (≥3.09)	9300	48,479	96	**1.55 (1.10, 2.19)**	**1.43 (1.01, 2.03)**
*p*_trend_				**0.008**	**0.031**
Continuous (log_2_)				**1.20 (1.06, 1.37)**	1.14 (1.00, 1.29)
Absolute neutrophil count (K/ul)					
Categorical					
1st tertile (<3.90)	9247	52,280	69	1.00	1.00
2nd tertile (3.90 to <5.57)	9298	52,185	66	0.98 (0.70, 1.38)	1.08 (0.77, 1.52)
3rd tertile (≥5.57)	9289	49,330	68	1.03 (0.74, 1.44)	1.10 (0.78, 1.55)
*p*_trend_				0.851	0.584
Continuous (log_2_)				0.95 (0.77, 1.16)	0.99 (0.81, 1.21)
Absolute lymphocyte count (K/ul)					
Categorical					
1st tertile (<1.55)	9269	48,730	98	1.00	1.00
2nd tertile (1.55 to <2.15)	9285	52,935	67	0.80 (0.59, 1.10)	0.92 (0.67, 1.26)
3rd tertile (≥2.15)	9280	52,130	38	**0.53 (0.36, 0.78)**	**0.64 (0.43, 0.94)**
*p*_trend_				**0.001**	**0.028**
Continuous (log_2_)				**0.66 (0.55, 0.78)**	**0.76 (0.63, 0.91)**

^a^ Adjusted for age and sex.

^b^ Adjusted for age, sex, history of type 2 diabetes, race, BMI category, smoking status, hyperlipidemia, hypertension, and FIB‐4 score category. Hazard ratios (HRs) with 95% confidence intervals (CIs) excluding one and *p* < 0.05 are in bold.

When stratified by age and sex, absolute lymphocyte count remained significantly associated with HCC among all subgroups (Table 4). Doubling of NLR and absolute lymphocyte count were both significantly associated with HCC risk (where doubling of NLR was associated with higher risk and doubling of lymphocyte count was associated with lower risk) among those with high BMI (30+ kg/m^2^) but not among those with low BMI (<30 kg/m^2^). The interaction term for this effect with BMI was significant only for absolute lymphocyte count (Table 4). Doubling of absolute lymphocyte count was associated with lower risk of HCC among those with a history of type II diabetes but not among those without. When stratified by cirrhosis diagnosis or FIB‐4 score category, doubling of NLR and absolute lymphocyte count were significantly associated with higher and lower HCC risk, respectively, only among NAFLD patients with low fibrosis score or without cirrhosis diagnosis. There was a significant interaction in the NLR‐HCC risk association between NAFLD patients with and without cirrhosis. To minimize the potential confounding effect of advanced fibrosis or cirrhosis, we further conducted sensitivity analysis after excluding individuals with cirrhosis or FIB‐4 ≥ 1.30 (for those <65 years) or FIB‐4 ≥ 2.0 (for those ≥65 years). This sensitivity analysis included 47 (approximately 20% of total) HCC cases among 16,889 NAFLD patients free of cirrhosis and FIB‐4 < 1.3/2.0 and produced consistent results that higher risk of HCC was associated with doubling NLR (HR = 1.36, 95% CI 1.03, 1.80) and lower risk of HCC was associated with doubling lymphocyte counts (HR = 0.61, 95% CI 0.40, 0.94), (data not shown).

**TABLE 4:** Association between neutrophil‐lymphocyte ratio (NLR) and the risk of hepatocellular carcinoma stratified by sex, age, BMI, history of type II diabetes, cirrhosis, and FIB‐4 score, The UPMC NAFLD Cohort Study, 2004–2018

Stratification variable	Cases/*N*	Neutrophil‐lymphocyte ratio	*p*	Absolute neutrophil count (K/ul)	*p*	Absolute lymphocyte count (K/ul)	*p*
Sex							
Female	96/15,807						
HR^c^ (95% CI)		1.13 (0.93, 1.38)	0.220	0.95 (0.70, 1.28)	0.723	**0.73 (0.55, 0.97)**	**0.036**
Male	107/12,027						
HR^c^ (95% CI)		1.14 (0.96, 1.35)	0.139	1.02 (0.77, 1.34)	0.916	**0.77 (0.61, 0.99)**	**0.041**
*p*_interaction_		0.545		0.733		0.573	
Age							
<60	57/16,857						
HR^c^ (95% CI)		**1.38 (1.09, 1.75)**	**0.008**	1.14 (0.77, 1.69)	0.520	**0.57 (0.40, 0.79)**	**<0.001**
60+	146/10,977						
HR^c^ (95% CI)		1.12 (0.96, 1.31)	0.149	0.85 (0.67, 1.08)	0.187	**0.68 (0.55, 0.85)**	**<0.001**
*p*_interaction_		0.178		0.241		0.358	
BMI (kg/m^2^)^a^							
<30	89/8760						
HR^c^ (95% CI)		1.10 (0.91, 1.33)	0.342	1.19 (0.88, 1.61)	0.270	0.95 (0.71, 1.27)	0.731
30+	101/18,037						
HR^c^ (95% CI)		**1.26 (1.05, 1.52)**	**0.012**	0.99 (0.74, 1.34)	0.964	**0.62 (0.48, 0.80)**	**<0.001**
*p*_interaction_		0.183		0.419		**0.009**	
History of Type II Diabetes							
No	140/20,478						
HR^c^ (95% CI)		1.15 (0.99, 1.34)	0.077	1.12 (0.88, 1.43)	0.350	0.82 (0.65, 1.04)	0.094
Yes	63/7356						
HR^c^ (95% CI)		1.12 (0.89, 1.41)	0.335	0.73 (0.50, 1.06)	0.102	**0.64 (0.48, 0.87)**	**0.004**
*p*_interaction_		0.984		0.177		0.360	
Cirrhosis							
No	69/24,372						
HR^c^ (95% CI)		**1.37 (1.10, 1.70)**	**0.006**	1.39 (0.97, 2.00)	0.070	**0.66 (0.47, 0.94)**	**0.020**
Yes	134/3462						
HR^c^ (95% CI)		0.97 (0.83, 1.14)	0.745	0.89 (0.70, 1.13)	0.332	0.95 (0.77, 1.19)	0.674
*p*_interaction_		**0.032**		0.053		0.153	
FIB‐4 score (age < 65/age ≥ 65)^b^							
<1.30/<2.00	90/18,445						
HR^c^ (95% CI)		**1.29 (1.05, 1.58)**	**0.014**	1.30 (0.95, 1.80)	0.106	**0.72 (0.53, 0.99)**	**0.040**
1.30–2.67/2.00–2.67	45/5141						
HR^c^ (95% CI)		1.01 (0.76, 1.36)	0.930	1.00 (0.64, 1.57)	0.990	0.97 (0.63, 1.51)	0.906
>2.67	51/2278						
HR^c^ (95% CI)		1.02 (0.82, 1.27)	0.874	0.75 (0.53, 1.04)	0.084	0.77 (0.57, 1.03)	0.079
*p*_interaction_		0.239		0.065		0.686	

^a^ Excluding 1037 patients with missing BMI.

^b^ Excluding 1970 patients with missing FIB‐4 score.

^c^ Hazard Ratio (95% CI) for continuous (log_2_) of white blood cell measurement or ratio. Adjusted for age, sex, history of type 2 diabetes, race, BMI category, smoking status, hyperlipidemia, hypertension, and FIB‐4 score category, if applicable. Models stratified by age were not adjusted for FIB‐4 score as age is a component of FIB‐4. Hazard ratios (HRs) with 95% confidence intervals (CIs) excluding one and *p* < 0.05 are in bold.

When stratified by follow‐up time, higher absolute lymphocyte count was significantly associated with lower risk of HCC only among NAFLD patients with less than 2 years of follow‐up (HR for 3rd versus 1st tertile, 0.43 95% CI: 0.24–0.76) (Table S3). The risk of HCC was reduced by a statistically significant 32% with the doubling of absolute lymphocyte count in patients with <2 years of follow‐up (Table S3), and in those with <1 year of follow‐up as well (data not shown). The lack of statistically significant association for NLR and lymphocyte count with HCC risk among those with 2 or more years of follow‐up might be due to fewer HCC cases, however we also saw no association among those with 1 or more years of follow‐up (data not shown).

We also conducted analysis for repeated measurements of white blood cells and risk of HCC, which might reflect a long‐term NLR or lymphocyte count for a given individual, although not all patients had repeated measures. The associations for the repeated NLR or absolute lymphocyte count with risk of HCC became stronger than those for the baseline measurement only. The multivariable‐adjusted HR of HCC for the highest tertile of NLR was 1.91 (95% CI: 1.30, 2.82) compared with the lowest tertile, and the corresponding figures for absolute lymphocyte count was 0.54 (95% CI: 0.36, 0.81) (Table S4). The null association remained for repeated measurement of absolute neutrophil count.

## 4 DISCUSSION

In this retrospective cohort of NAFLD patients, we found that both baseline and repeated measurements of lower absolute lymphocyte count and higher NLR were associated with a statistically significant higher risk of HCC among NAFLD patients without viral hepatitis, alcohol disorder, or other major established causes of underlying liver disease. We found no association between absolute neutrophil count and HCC risk, suggesting that the association between NLR and HCC risk is driven by absolute lymphocyte count. We also found that absolute lymphocyte count was associated with HCC risk among those with high BMI and NLR was significantly associated with HCC risk among those without cirrhosis diagnosis, where both associations had significant interaction (*p*_interaction_ <0.05). Both BMI and cirrhosis are major risk factors for HCC,^4^ which may mask the effect of NLR on HCC risk. Furthermore, the observed significant association between NLR (or lymphocyte count) and HCC risk among NAFLD patients without advanced fibrosis (i.e., FIB‐4 score <1.30 for <65 years or <2.0 for 65+ years old) suggests that these biomarkers are more likely to be an early marker for HCC, rather than biomarkers for unidentified cirrhosis. The association between NLR, absolute lymphocyte count, and HCC risk remained significant only among those with less than 2 years of follow‐up, suggesting that low absolute lymphocyte count may be a marker of early cancer rather than an independent risk factor. However, due to the small sample size among those with more than 2 years of follow‐up, future studies are warranted to investigate the role of NLR and absolute lymphocyte count in prospective cohorts of NAFLD patients with longer follow‐up.

NLR is a common prognostic marker for HCC. Different studies have found an association between higher levels of pre‐treatment NLR and poor prognosis in HCC patients such as higher cancer recurrence and metastasis, shorter overall survival, and graft survival after liver tranplantation.^29^, ^30^, ^31^ The potential mechanism may be that patients with low lymphocyte count may have reduced immune surveillance capacity to inhibit tumor cell growth or migration through either cytotoxic activity or cytokine production, or through high infiltration of tumor‐associated macrophages (TAMs) and high production of inflammatory cytokines (Interleukin [IL]‐6, IL‐8, IL‐17), which stimulate neutrophilia.^31^, ^32^, ^33^ However, few studies have examined NLR as a marker for risk of HCC development in the NAFLD populations. One prospective cohort study in China reported a high NLR level was associated with a higher risk of HCC among individuals who had cirrhosis caused by HBV.^34^ Another retrospective cohort in China found that higher NLR was associated with higher HCC risk in HBV‐cirrhotic patients after splenectomy.^35^ Another study in the UK found that HCC cases had statistically significantly higher NLR at baseline compared to control patients with chronic liver disease.^36^ Despite the differences in study populations and study design, epidemiological studies between NLR and HCC without HBV or HCV infection are sparse. To our knowledge, our study may be the first attempt to examine pre‐diagnostic lymphocyte counts and NLR and the risk of HCC development in a cohort of NAFLD patients without viral hepatitis or other major risk factors for underlying liver diseases.

The immune system plays a critical role in controlling cancer growth and immune surveillance is critical for the control of neoplastic development.^37^, ^38^ Individuals with immunodeficiency may be at higher risk of HCC.^39^ In the US HIV/AIDS Cancer Match Study (over 610,000 individuals who had AIDS in the US), the risk of HCC was found to be 4 times higher than the general population.^39^ The higher risk of HCC may be driven by the immunosuppressive effect of HIV that results in lower lymphocyte counts and reduced immune surveillance.^40^, ^41^ Lymphocytes consist of natural killer cells, T cells, and B cells, and are the key players in human adaptive immunity. Lymphocytes produce important cytokines such as TNF‐α, inhibiting tumor growth.^42^, ^43^ Tumor antigens that are produced by genetic variations can also be differentiated by adaptive immunity.^44^ Natural killer cells contributed to the lysis of HCC in vitro by recognizing the expression of the Major Histocompatibility Complex Class I polypeptide‐related sequence A gene, which is commonly expressed in liver cancers.^45^, ^46^ Therefore, lymphopenia or lymphocytopenia, may reduce the adaptive immune responses and increase the risk of cancer such as HCC.^47^ In addition, mouse models suggest that NASH may suppress CD4 and CD8 T cells which may promote hepatocarcinogenesis through reduced immune surveillance.^48^ Further studies are needed to understand the biologic mechanism for the link between NLR and HCC risk, and a potential role of systemic immune surveillance by lymphocytes.

Our study has several strengths. We defined a cohort of more than 27,000 NAFLD patients with pre‐diagnostic measurement of blood neutrophil and lymphocyte counts who were free of major chronic liver diseases such as HBV/HCV, alcoholic liver disease, and auto‐immune disease. Additionally, while our cohort design was retrospective, our analysis was prospective between lymphocyte measurement and HCC risk because the measurements of all lymphocyte, neutrophil, and white blood cell count in circulation were done prior to HCC diagnosis, which reduced the potential impact of HCC disease progression, diagnostic procedures and/or treatment on the neutrophil and lymphocyte counts. We were also able to use repeated measurements available to show consistent results with HCC risk with baseline measurement of the biomarkers, suggesting a robust effect of NLR or lymphocyte count on the disease risk. Our results in a representative sample of NAFLD patients in Western Pennsylvania may be generalizable to the general population in the United States.

Our study also has several limitations. The administrative dataset of patients' EHRs used might be subject to incomplete diagnosis and/or ICD coding for the NAFLD patients. Although UPMC is the largest healthcare provider in western Pennsylvania, some patients with NAFLD might not participate in this health plan and thus were not included in the present study, which might result in a selection bias. We excluded approximately 30% of the original NAFLD patients because of lack of white blood cell measurements, which might introduce a selection bias on our observed results. There may be potential residual confounding effects on the observed associations between NLR and HCC such as low level of alcoholic drinking status, which may not completely be recorded in the EHR even though alcoholic disorders are excluded from the study population. Platelet count, AST, ALT, BMI, and smoking status were all selected based on the nearest date available to white blood cell measurements to maximize sample size, which may introduce variability and misclassification in these variables. Lack of information on other lifestyle factors such as lower consumption of alcohol, physical inactivity, and unhealthy dietary habits might also lead to potential residual confounding on the NLR‐HCC risk association, although there is not strong evidence supporting the impact of these lifestyle factors on the NLR value. Finally, the incidence of HCC in NAFLD patients was low which limited the statistical power in our stratified analyses.

In conclusion, in a large cohort study of 27,834 NAFLD patients, we found that lower absolute lymphocyte count and higher NLR were associated with a higher risk of HCC development. These findings warrant further investigation of systemic immune surveillance by lymphocytes in association with HCC development in patients with NAFLD.

## AUTHOR CONTRIBUTIONS

Study Conception and Design: Claire E. Thomas, Jian‐Min Yuan, Renwei Wang. Acquisition of Data: Jian‐Min Yuan. Analysis and Interpretation of Data: Jian‐Min Yuan, Renwei Wang, Claire E. Thomas, Drafting of the Manuscript: Claire E. Thomas, Yi‐Chuan Yu. Critical Revision of the Manuscript: All authors. Administrative, Technical, or Material Support: Jian‐Min Yuan. Study Supervision: Jian‐Min Yuan.

## FUNDING INFORMATION

This research project was partially supported by an US NIH grant (No. R01CA255809 to J. Behari and J.‐M. Yuan), the University of Pittsburgh Medical Center (UPMC) Hillman Cancer Center Start‐up fundings (to H.N. Luu and J.‐M. Yuan). C.E. Thomas and P Paragomi were supported by the NIH T32CA186873 (PI: J‐M Yuan) training grant in cancer epidemiology and prevention.

## CONFLICT OF INTEREST

No conflict of interest was declared by any other authors.

## INFORMED CONSENT & IRB APPROVAL

The University of Pittsburgh Internal Review Board (IRB) has approved the request for the de‐identified electronic health records (EHRs). Informed consent was not obtained because de‐identified medical records were requested through the Health Record Research Requestion (R3) provided by the Biomedical Informatics Services at the University of Pittsburgh.

## Supporting information


Table S1
Table S2
Table S3
Table S4

## Data Availability

The data that support the findings of this study are available from the corresponding author upon reasonable request.
