# Supplementary material for: Neutrophil‐lymphocyte ratio in relation to risk of hepatocellular carcinoma in patients with non‐alcoholic fatty liver disease
Source: Cancer Med. 2022 Sep 2;12(3):3589–600. doi: 10.1002/cam4.5185 (PMC9939106; doi:10.1002/cam4.5185)
Supplement: Supplementary file 1 — Table S1 Table S2 Table S3 Table S4 [file CAM4-12-3589-s001.docx]

**Supplementary** **Tables & Figures: Neutrophil-lymphocyte ratio in relation to risk of hepatocellular carcinoma in patients with non-alcoholic fatty liver disease**

| **Supplementary Table 1.** The list of ICD codes as inclusion criteria for the UPMC NAFLD Cohort Study, 2004-2018   \| **Disease Name** \| **ICD Code** \| **Code Type** \| **Descriptions** \| \| --- \| --- \| --- \| --- \| \| Non-alcoholic Fatty Liver \| 571.8 \| ICD9CM \| Other chronic nonalcoholic liver disease \| \| K76.0 \| ICD10CM \| Fatty (change of) liver, not elsewhere classified \| \| Nonalcoholic Steatohepatitis \| K75.8 \| ICD10CM \| Other specified inflammatory liver diseases \| \| Compensated Cirrhosis \| 571.5 \| ICD9CM \| Cirrhosis, compensated \| \| K74.6 \| ICD10CM \| Cirrhosis, compensated \| \| 456.1 456.21 \| ICD9CM \| Esophageal varices, not bleeding \| \| I85.9  I98.2 \| ICD10CM \| Esophageal varices, not bleeding \| \| I86.4 \| ICD10CM \| Gastric varices, not bleeding \| \| Decompensated Cirrhosis \| 456.0  456.20 \| ICD9CM \| Esophageal varices, bleeding \| \| I85.0  I98.3 \| ICD10CM \| Esophageal varices, bleeding \| \| 789.5 \| ICD9CM \| Ascites \| \| R18 \| ICD10CM \| Ascites \| \| 572.2 \| ICD9CM \| Hepatic encephalopathy \| \| 572.4 \| ICD9CM \| Hepatorenal syndrome \| \| K76.7 \| ICD10CM \| Hepatorenal syndrome \| \| 572.3 \| ICD9CM \| Portal hypertension \| \| K76.6 \| ICD10CM \| Portal hypertension \|   **Supplementary Table 2.** The list of ICD codes as exclusion criteria for the UPMC NAFLD Cohort Study, 2004-2018   \| **Disease Name** \| **ICD9CM** \| **ICD10CM** \| \| --- \| --- \| --- \| \| Alcoholic liver disease \| 571.0-571.3 \| K70 \| \| Alcohol use disorder \| 303, 305.0 \| F10 \| \| Somatic consequences of alcohol (except alcoholic liver disease) \| 291, 357.5, 425.5, 535.3, 980.1, 980.9 \| E24.4, G62.1, I42.6, K29.2, G31.2, G72.1, K85.2, K86.0, T51.0, T51.9, Y57.3, X65, Z50.2, Z71.4, Z72.1 \| \| Autoimmune liver diseases \| 571.6, 576.1 \| K83.0A, K83.0F, K74.3, K75.4 \| \| Alpha-1-antitrypsin deficiency \| 277.6 \| E88.0A, E88.0B \| \| Secondary or unspecified biliary cirrhosis \| 571.6 \| K74.4, K74.5 \| \| Drug use disorders except nicotine/caffeine \| 305.1-305.9 \| F11-F14, F16, F18, F19 \| \| Hemochromatosis \| 275.0 \| E83.1 \| \| Budd-Chiari syndrome \| 453.0 \| I82.0, K76.5 \| \| Viral hepatitis \| 70 \| B16, B17, B18, B19 \| \| Chronic hepatitis, unspecified \| 571.4 \| K73.9, K73.2 \| \| Wilson’s disease \| 275.1 \| E83.0B \| |  |  |  |  |
| --- | --- | --- | --- | --- | --- | --- | --- | --- | --- | --- | --- | --- | --- | --- | --- | --- | --- | --- | --- | --- | --- | --- | --- | --- | --- | --- | --- | --- | --- | --- | --- | --- | --- | --- | --- | --- | --- | --- | --- | --- | --- | --- | --- | --- | --- | --- | --- | --- | --- | --- | --- | --- | --- | --- | --- | --- | --- | --- | --- | --- | --- | --- | --- | --- | --- | --- | --- | --- | --- | --- | --- | --- | --- | --- | --- | --- | --- | --- | --- | --- | --- | --- | --- | --- | --- | --- | --- | --- | --- | --- | --- | --- | --- | --- | --- | --- | --- | --- | --- | --- | --- | --- |

**Supplementary Table 3. Association between neutrophil-lymphocyte ratio, absolute neutrophil count, and absolute lymphocyte count and the risk of hepatocellular carcinoma stratified by follow-up time,**

**The UPMC NAFLD Cohort Study, 2004 – 2018**

|  | **≤2 Years of Follow-Up** | | |  | **>2 Years of Follow-Up** | | |
| --- | --- | --- | --- | --- | --- | --- | --- |
| **Exposure** | **Total No.**  **Subjects** | **HCC Cases** | **HR (95% CI)*** |  | **Total No.**  **Subjects** | **HCC Cases** | **HR (95% CI)*** |
| **Total** | 27,834 | 113 | - |  | 22,858 | 90 | - |
| **Neutrophil-lymphocyte ratio** |  |  |  |  |  |  |  |
| Categorical |  |  |  |  |  |  |  |
| 1^st^ tertile (<1.97) | 9260 | 24 | 1.00 |  | 7891 | 27 | 1.00 |
| 2^nd^ tertile (1.97-<3.09) | 9274 | 35 | 1.36 (0.81, 2.30) |  | 7829 | 21 | 0.73 (0.41, 1.30) |
| 3^rd^ tertile (≥3.09) | 9300 | 54 | 1.58 (0.97, 2.60) |  | 7138 | 42 | 1.30 (.80, 2.14) |
| *P_trend_* |  |  | 0.072 |  |  |  | 0.224 |
| Continuous (log_2_) |  |  | 1.17 (0.99, 1.39) |  |  |  | 1.10 (0.89, 1.35) |
| **Absolute neutrophil count (K/uL)** |  |  |  |  |  |  |  |
| Categorical |  |  |  |  |  |  |  |
| 1^st^ tertile (<3.90) | 9247 | 41 | 1.00 |  | 7762 | 28 | 1.00 |
| 2^nd^ tertile (3.90-<5.57) | 9298 | 38 | 1.08 (0.69, 1.69) |  | 7746 | 28 | 1.09 (0.64, 1.85) |
| 3^rd^ tertile (≥5.57) | 9289 | 34 | 0.94 (0.59, 1.50) |  | 7350 | 34 | 1.35 (0.81, 2.27) |
| *P_trend_* |  |  | 0.822 |  |  |  | 0.245 |
| Continuous (log_2_) |  |  | 0.93 (0.72, 1.21) |  |  |  | 1.10 (0.80, 1.51) |
| **Absolute lymphocyte count (K/uL)** |  |  |  |  |  |  |  |
| Categorical |  |  |  |  |  |  |  |
| 1^st^ tertile (<1.55) | 9269 | 63 | 1.00 |  | 7177 | 35 | 1.00 |
| 2^nd^ tertile (1.55-<2.15) | 9285 | 35 | 0.82 (0.53, 1.25) |  | 7824 | 32 | 1.10 (0.67, 1.78) |
| 3^rd^ tertile (≥2.15) | 9280 | 15 | **0.43 (0.24, 0.76)** |  | 7857 | 23 | 0.97 (0.56, 1.67) |
| *P_trend_* |  |  | **0.005** |  |  |  | 0.945 |
| Continuous (log_2_) |  |  | **0.68 (0.54, 0.86)** |  |  |  | 0.90 (0.66, 1.22) |

*Adjusted for age, sex, history of type 2 diabetes, race, BMI category, smoking status, hyperlipidemia, hypertension, and FIB-4 score category.

Hazard ratios (HRs) with 95% confidence intervals (CIs) excluding one and *P* < 0.05 are in bold.

**Supplementary Table 4. Time-varying Cox proportional hazard model for the association between neutrophil-lymphocyte ratio, absolute neutrophil count, and absolute lymphocyte count and the risk of hepatocellular carcinoma, The UPMC NAFLD Cohort Study, 2004 – 2018**

| **Exposure** |  | **Total follow-up time for repeated measurements (years)** | **HCC Cases** | **HR (95% CI)^1^** | **HR (95% CI)^2^** |
| --- | --- | --- | --- | --- | --- |
| **Neutrophil-lymphocyte ratio** | | | | | |
| Categorical |  |  |  |  |  |
| 1^st^ tertile (<1.97) |  | 43,849 | 34 | 1.00 | 1.00 |
| 2^nd^ tertile (1.97-<3.09) |  | 51,098 | 43 | 1.02 (0.65, 1.60) | 1.04 (0.66, 1.63) |
| 3^rd^ tertile (≥3.09) |  | 58,848 | 126 | **2.03 (1.38, 2.98)** | **1.91 (1.30, 2.82)** |
| *P_trend_* |  |  |  | **<0.001** | **<0.001** |
| Continuous (log_2_) |  |  |  | **1.43 (1.25, 1.63)** | **1.36 (1.19, 1.56)** |
| **Absolute neutrophil count (K/uL)** | | | | | |
| Categorical |  |  |  |  |  |
| 1^st^ tertile (<3.90) |  | 46,789 | 62 | 1.00 | 1.00 |
| 2^nd^ tertile (3.90-<5.57) |  | 55,059 | 62 | 0.85 (0.60, 1.21) | 0.96 (0.67, 1.37) |
| 3^rd^ tertile (≥5.57) |  | 51,947 | 79 | 1.08 (0.78, 1.51) | 1.20 (0.85, 1.69) |
| *P_trend_* |  |  |  | 0.581 | 0.270 |
| Continuous (log_2_) |  |  |  | 1.11 (0.95, 1.30) | 1.14 (0.98, 1.32) |
| **Absolute lymphocyte count (K/uL)** | | | | | |
| Categorical |  |  |  |  |  |
| 1^st^ tertile (<1.55) |  | 50,148 | 112 | 1.00 | 1.00 |
| 2^nd^ tertile (1.55-<2.15) |  | 54,527 | 59 | **0.65 (0.47, 0.89)** | 0.75 (0.54, 1.03) |
| 3^rd^ tertile (≥2.15) |  | 49,119 | 32 | **0.46 (0.31, 0.68)** | **0.54 (0.36, 0.81)** |
| *P_trend_* |  |  |  | **<0.001** | **0.002** |
| Continuous (log_2_) |  |  |  | **0.58 (0.48, 0.71)** | **0.68 (0.55, 0.83)** |

^1^ Adjusted for age and sex.

^2^ Adjusted for age, sex, history of type 2 diabetes, race, BMI category, smoking status, hyperlipidemia, hypertension, and FIB-4 score category. Hazard ratios (HRs) with 95% confidence intervals (CIs) excluding one and *P* < 0.05 are in bold.
